# Evolutionary aspects of the Viridiplantae nitroreductases

**DOI:** 10.1186/s43141-020-00073-3

**Published:** 2020-10-06

**Authors:** Siarhei A. Dabravolski

**Affiliations:** Department of Clinical Diagnostics, Vitebsk State Academy of Veterinary Medicine [UO VGAVM], 7/11 Dovatora St., 210026 Vitebsk, Belarus

**Keywords:** Nitroreductase, Viridiplantae, Cyanobacteria, Phylogeny, Evolution

## Abstract

**Background:**

Nitroreductases are a family of evolutionarily related proteins catalyzing the reduction of nitro-substituted compounds. Nitroreductases are widespread enzymes, but nearly all modern research and practical application have been concentrated on the bacterial proteins, mainly nitroreductases of *Escherichia coli*. The main aim of this study is to describe the phylogenic distribution of the nitroreductases in the photosynthetic eukaryotes (Viridiplantae) to highlight their structural similarity and areas for future research and application.

**Results:**

This study suggests that homologs of nitroreductase proteins are widely presented also in Viridiplantae. Maximum likelihood phylogenetic tree reconstruction method and comparison of the structural models suggest close evolutional relation between cyanobacterial and Viridiplantae nitroreductases.

**Conclusions:**

This study provides the first attempt to understand the evolution of nitroreductase protein family in Viridiplantae. Our phylogeny estimation and preservation of the chloroplasts/mitochondrial localization indicate the evolutional origin of the plant nitroreductases from the cyanobacterial endosymbiont. A defined high level of the similarity on the structural level suggests conservancy also for the functions. Directions for the future research and industrial application of the Viridiplantae nitroreductases are discussed.

## Background

Nitroreductases are a family of closely related proteins that catalyze the reduction of nitro-substituted compounds, using FMN (Flavin mononucleotide) or FAD (Flavin adenine dinucleotide) as a cofactor and NADH (Nicotinamide adenine dinucleotide) or NADPH (Nicotinamide adenine dinucleotide phosphate) as a reducing agent. Nitroreductases are ancient enzymes, with approximate evolutionary age ~ 2.5 billion years and represented by more than 26,000 known sequences [19]. In addition to the nitroreduction reaction, nitroreductases are known to catalyze a wide range of other reactions (dehalogenation, dehydrogenation, flavin fragmentation) and apply a wide range of substrates (metal ions, quinone, flavin, nitroaromatic, and enone compounds) (reviewed in [61]). While nitroreductase enzymes are widespread and their reactions diversity is well-characterized, nearly all modern research for has been concentrated on the proteins of *Escherichia coli*. Thus, elucidating the evolutionary relations of the plant nitroreductases could facilitate their further research and industrial application.

Nitroreductases have high potential in their utility in activating prodrugs in directed anticancer therapies (reviewed in [61]. Bacterial nitroreductase NfsB (*Escherichia coli*) was applied with positive clinical outcomes for the treatment of prostate cancer and brain tumors [45]. Recently, *Mycobacterium smegmatis* nitroreductase NfnB was used as a pharmaceutical and chemicals synthesis agent to obtain new compound BTZ043 for the treatment of tuberculosis [37]. To bioremediate and degrade the world-wide use pollutant polychlorinated biphenyl was successfully created transgenic tobacco plants, expressing nitroreductase *bphC* gene from *Pandoraea pnomenusa* [44]. Also, nitroreductase NfsA from *Escherichia coli* was successfully used in the biocatalysis of several nitroaromatic compounds and quinones [56].

The current classification includes two classes of nitroreductases: type I (oxygen-insensitive) catalyze the reduction of organic nitro compounds using a two-electron transfer mechanism to primary amines [31] and type II (oxygen-sensitive) catalyzes a one-electron reduction of the nitro group to produce nitro anion radicals that may react with oxygen, form superoxide and cause oxidative stress [48]. In the yeast, *Saccharomyces cerevisiae*, 2 genes, frm2 (YCL026c-A) (fatty acid repression mutant) and hbn1 (YCL026c-B) (homologous to bacterial nitroreductases), encoding putative nitroreductase-like proteins were identified by in silico analysis [15]. The biological functions of the yeast nitroreductase family of proteins are not well studied; however, their possible involvement in oxidative stress responses has been suggested [3]. Experimental data on Frm2 protein indicate that Frm2 may be involved in the lipid signalling pathway and cellular homeostasis [40]. Also, solved crystal structure [52] supports this finding and provides insights into the molecular mechanism of the yeast Frm2 activity.

Human DEHAL1 (Iodotyrosine dehalogenase 1) is a well-characterized member of the nitroreductase family responsible for iodide recycle [26] and thyroid hormone synthesis [8]. It was shown that nitroreductase and dehalogenase activities are closely related to the sequence level [43].

The recent advantage in the sequencing technologies and genes annotation with an automatic pipeline allows identifying many genes as “nitroreductase family member” in Viridiplantae. Despite the undoubted importance of the nitroreductases, their characterization in Viridiplantae is missing. Thus, although a recent study has provided a deep insight into the understanding of the evolution of nitroreductases [1], the evolutional history of the green lineage nitroreductases has not been addressed as broadly as in other kingdoms.

In the present work, all currently available genomic resources were used to explore the diversity and the phylogenetic distribution of the nitroreductase domain-containing proteins in Viridiplantae. This study represents the first step toward understanding the evolution of the nitroreductase proteins in the green lineage. Altogether, results of this study could facilitate further research and industrial application of the Viridiplantae nitroreductases.

## Methods

### Identification of the nitroreductases in the Viridiplantae clade

Nitroreductases were identified with keyword search and following BLAST (Basic local alignment search tool) [51] searches in NCBI (National Center for Biotechnology Information), InterPro 77 [42], Pfam 32.0 [19], and Phytozome 12.1 [21] databases. The consensus sequence of the nitroreductase domain (PF00881/IPR029479) was used in on-line BLASTP (Basic Local Alignment Search Tool Protein) searches. All partial and fragmented sequences were eliminated. Presence of the nitroreductase domain was checked with CD-search (NCBI) [38] and MOTIF search (KEGG 93) [30] tools with E-value (≤ 0.001). Domains, fused to the nitroreductase domain, were verified with the same tools and threshold.

### Multiple sequence alignments and phylogenetic analysis

Nitroreductase domain sequences were extracted from Pfam database [19] (for proteins with 2 nitroreductase domains, the sequence of N-terminal one was used) and multiple sequence alignments were performed with MUSCLE [18]. The test of substitution models and phylogenetic analysis were carried out using the MEGA X software [32]. For maximum likelihood tree [60], the LG substitution model [34] was selected assuming an estimated proportion of invariant sites and 4 gamma-distributed rate categories to account for rate heterogeneity across sites. The gamma shape parameter was estimated directly from the data. Reliability for the internal branch was assessed using the bootstrapping method (1000 bootstrap replicates). The same settings with the JTT substitution model [28] were used for reconstruction with the Neighbor-Joining [50] method.

### Localization prediction and structure modeling

Subcellular localization was predicted with TargetP on-line tool [20], “Plant Organism group” settings were used. Model of *Arabidopsis* nitroreductase (At1G02020) was built with SWISS-MODEL [59] and matched with *Anabaena variabilis* nitroreductase (PDB 3EO7 (10.2210/pdb3EO7/pdb)) in Chimera software [49]. Quality of the created models was verified with QMEAN [5]. VAST+ was used to search for the structure similarity [36], iPBA webserver was used for the pdb structures alignment (https://www.dsimb.inserm.fr/dsimb_tools/ipba/index.php). The quality of the structures alignments was evaluated with RMSD and normalized score [55, 64].

## Results

### Exploring the distribution of nitroreductases in Viridiplantae

All currently available Viridiplantae species genomes were checked, and 97 proteins containing nitroreductase domain (Supplementary Table 1) were identified. Most nitroreductases are single-domain proteins, containing only nitroreductase domain, but there are some examples of nitroreductases with doubled nitroreductase domain. In 13 proteins, this additional C-terminal domain was significantly above threshold (designated as ×2 in Supplementary Table 1), in all other cases, the domain was partial and below a defined threshold. Two proteins (D7KP50 and A0A2P5WXT2) have N-terminal C2H2-type zinc finger (in D7KP50) and Myb/SANT-like DNA-binding (in A0A2P5WXT2) domains (from *Arabidopsis lyrata* L. and *Gossypium barbadense* L., respectively) that may suggest their additional function as transcription factors.

Several recent studies have provided important advances in our understanding of the structure and distribution of nitroreductases in many species: Bacteria, Fungi (Oliveira 2007); yeast (*Saccharomyces cerevisiae*) (Song 2015); *Clostridium difficile* (Wang [57]); nematode *Caenorhabditis elegans* [14]; mouse (*Mus musculus*) [54]; human [25]. However, phylogenetic analysis of nitroreductases in the whole green lineage (Viridiplantae) is missing.

Genome searches revealed that nitroreductases are present in the Chlorophyta (green algae) (11 proteins), mosses (1 protein in *Physcomitrella patens* Hedw [33]., liverwort (1 protein in *Marchantia polymorpha* L. [10], clubmosses (2 proteins in *Selaginella moellendorffii* [4]. No nitroreductases were found in Red Algae. Also, nitroreductase proteins were found in Acrogymnospermae: *Wollemia nobilis* [47, 62] and *Araucaria cunninghamii Mudie.* (one protein in each). Ancient flowering plant *Amborella trichopoda Baill* [2]., *Cinnamomum micranthum* (Hayata) [13], and *Macleaya cordata Willd* [35]. were containing one nitroreductase protein each, but three proteins were found in *Nelumbo nucifera Gaertn* [41]. Monocotyledons and eudicotyledons were represented with a higher number of proteins, 10 and 64 respectively (Supplementary Table 1).

To gain insight into the phylogenetic relationships between nitroreductases from green algae (Chlorophyta) and land plants (Streptophyta), the robust phylogenetic tree after multiple alignments of 100 predicted sequences (Supplementary Figure 1 and 2) was generated. Nitroreductases from Cyanobacteria (*Anabaena cylindrical* and *Nostoc punctiforme*) and Chloroflexi (*Chloroflexus islandicus*) were added as green photosynthetic bacteria outgroup. As expected, nitroreductases from Streptophyta and Green algae were clustered in separated branches, with closer relation of Bacterial nitroreductases to Green algae. Also, high homology on the level of the family that decreasing dramatically in higher taxa could be noticed. Thus, this represents the first description of nitroreductase homologs in Green Algae and Streptophyta.

### Structural models comparison

**Fig. 1:**
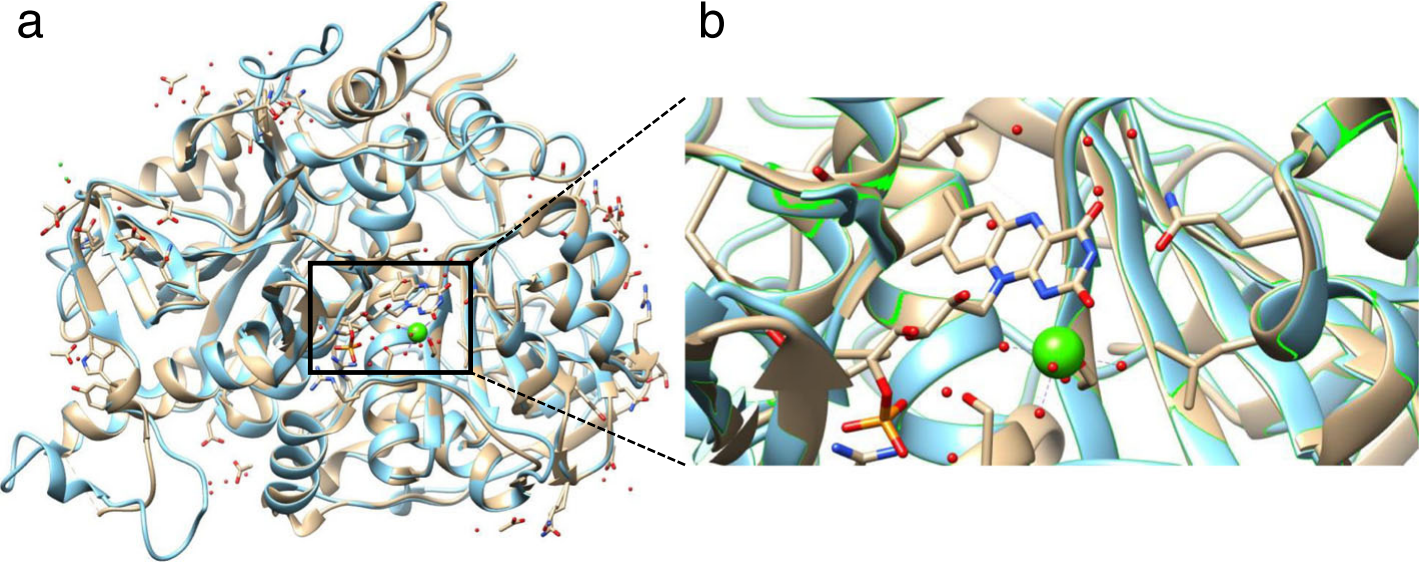
**a** Matched crystal structures of putative nitroreductase from *Arabidopsis thaliana* (blue) and *Anabaena variabilis* (PDB 3EO7) (pale pink). **b** Closer look at the FMN (in the centre) binding site and Ca^2+^ ion (green ball)

Structural alignment of the *Arabidopsis thaliana* chloroplasts/mitochondria-localized nitroreductase (O23673) with different known nitroreductases (Table 1) confirms results obtained with the phylogenetic tree. In comparison to different bacteria and yeast, cyanobacteria *Anabaena variabilis* shown the highest structural similarity to the nitroreductase from *Arabidopsis thaliana* (Fig. 1a)*.* It is important to notice, that amino acids, required for the co-factor (FMN) binding are conserved in cyanobacteria and Arabidopsis (Fig. 1b): 98R, 101P, 102S, 188D, 191H, and 334R. These features suggest that plant nitroreductases are most likely to perform functions, similar to bacterial. The main difference between plant and bacterial nitroreductases is the presence of the N-terminal peptide (1–40 in O23673), required for the protein translocation to the chloroplasts and/or mitochondria.

**Table 1:** Structural alignment of the Arabidopsis O23673 protein (Locus At1g02020) with nitroreductases with defined structures from different species

Organism	PDB ID	Normalized score	RMSD	GDT TS
*Geobacter Metallireducens Gs-15*	4DN2	− 204.97	1.67	16.72
*Clostridium difficile R20291*	5J62	− 277.48	2.75	2.14
*Chlorobium tepidum*	2R01	− 231.45	1.71	15.53
*Ralstonia eutropha jmp134*	3HJ9	− 177.6	1.82	20.26
*Bacteroides fragilis NCTC 9343*	3EK3	− 313.01	2.67	2.73
*Vibrio parahaemolyticus RIMD 2210633*	5UU6	− 246.08	1.77	15.48
*Saccharomyces Cerevisiae*	4URP	− 230.67	2.4	13.71
*Anabaena variabilis atcc 29413*	3EO7	130.13	0.73	73.69

Based on the obtained phylogenetic results and predominant localization of the plant nitroreductases in chloroplasts, we could suggest the general line of evolution from cyanobacteria via endosymbiotic event to the modern chloroplasts. Overall, our assumption is well-supported by the modern theory of the chloroplasts and mitochondria origin from bacterial ancestors [39].

## Discussion

In this study, for the first time, predicted nitroreductases in unexplored eukaryotic Viridiplantae supergroup were described. As it was shown in previous studies [15, 22] nitroreductases have very low sequence identity/similarity. This fact may explain why their presence in Viridiplantae was overlooked. Based on the ancient nature of the nitroreductases (approximate evolutionary age ~ 2.5 billion years [58]), wide representation among different taxa, we assume that nitroreductases are omnipresent enzymes and also presented in Viridiplantae. Application of different BLAST search strategies allows to identify nitroreductases literally in all Viridiplantae species but mostly with partial nitroreductase domain or below a threshold value.

Up to date, not much is known about localization of the nitroreductase activity in mammalian cells. The recently developed fluorescent sensor allows to image sub-mitochondrial localization of nitroreductase activity in live HEK 293 cells (human embryonic kidney cells) [53]. Similarly, bacterial nitroreductases, transformed into plants, have higher activity in case of chloroplast and mitochondrial localization [65]. Most probably such localization is connected to the role in oxidative stress response and regulation of antioxidant enzymes as it was shown in yeast [3, 16] and detoxification of the photosynthesis by-products [7]. Predicted localization of the defined plant nitroreductases corresponds with this assumption, and majority of proteins were predicted to have chloroplast and/or mitochondrial localization (Supplementary Table 1). The only exception with no organelle localization is the secreted nitroreductase (A0A0D2VDD8) from cotton *Gossypium raimondii* Ulbr.

Most probably, plant nitroreductases are participating in the oxidative stress, pollutant, and herbicide responses [7, 65]. Also, some connections to circadian rhythms or the efficiency of the photosynthetic machine are possible. In particular, by-products of many pollutant and herbicide are known to degrade in the mitochondria and cause the production of the highly toxic reactive superoxide. The reduction of the superoxide is catalyzsed by several families of closely related reductases localized in the mitochondria and chloroplasts, like, for example, monodehydroascorbate reductases [27] and quinone oxidoreductase [6]. Most probably, plant nitroreductases are also participating in the superoxide reduction. Also, it is known that some pollutants could damage photosynthetic apparatus and decrease the content of chlorophyll, but these negative effects are neutralized by the overexpression of transgenic bacterial nitroreductase [23], thus, suggesting photoprotective role.

The main focus of modern nitroreductases research is oriented on substrate recognition specificity, kinetic parameters related to prodrug activation or antibiotic resistance, but missing detailed characterization of regulatory mechanisms (reviewed by [61]). Based on available data, bacterial nitroreductases are induced by oxidative stress or decreases intracellular NAD(P)H to NAD(P)^+^ ratio [46]. Expression of the yeast nitroreductases is constitutive and does not depend on the cell physiological status [16]. Without any experimental data available, it is hard to predict regulatory mechanisms for the plant nitroreductases expression and functioning.

Plant nitroreductases have high potential in industrial application and biotechnology. It is known that substrate specificity of the nitroreductase could be changed just by the replacement of a single amino acid [43]. Thus, it is possible to adjust co-factor binding site, size, and shape of the substrate-binding pocket and create an enzyme suitable for degradation of the nearly any compound [17]. Bacterial enzymes are not much suitable for such purposes, because they lack membrane anchor and their localization is not specific. Partially, this problem could be solved by transplastomic transformation, providing production of the high amount of functional enzyme [65]. From the other side, this method has several limitations, mainly: (1) many pollutants are absorbed by roots, where chloroplastic enzymes are not presented, or their activity is very low [11]; (2) plastid genes are greatly downregulated in fruits, where pollutants are often concentrated [29]; in general, plastid transformation is well-established only in the limited number of species, but agricultural and industrial plants species are rather recalcitrant [9]. Further research in this area would allow engineering plant species resistant to herbicides and with target phytoremediation properties.

Interestingly, that comparison of homologs on the level of protein structures provided a possible evolutional relation of plant and bacterial nitroreductases (Table 1). Not surprisingly, the closest bacterial homolog of nitroreductase for *Arabidopsis thaliana* (L.) Heynh. (O23673) was nitroreductases from *Anabaena variabilis* (3eo7) (Fig. 1). Most probably that Viridiplantae has obtained nitroreductase via cyanobacterial endosymbiont [39].

Finally, this study reports the first description of the nitroreductases in the Viridiplantae supergroup. The low level of similarity between identified nitroreductases in Viridiplantae species complicated the phylogenomic analysis, and it was possible to make only a general overview of the evolutionary relationships of nitroreductases in this supergroup. Nitroreductase proteins have been thought to be absent from photosynthetic eukaryotes although this conclusion was made in rather old studies [12, 24] without application of modern similarity search algorithms and when a small number of Viridiplantae genomes was available.

Based on obtained results, some research directions for future investigation could be suggested: (1) proper classification of the Viridiplantae nitroreductase proteins based on their biochemical features (type I or II); (2) functional characterization of the newly defined proteins; 3) how localization of the nitroreductases (chloroplast, mitochondrial, or other) is related to the recognized substrates. Current attempts to create transgenic plants suitable for phytoremediation are based on the application of bacterial nitroreductases [7, 63, 65], although plant nitroreductases could provide better results. In addition to the application in the phytoremediation, transgenic plants, overexpressing nitroreductases could provide significant improvement during stress adaptation and disease resistance.

## Conclusions

The present study reports, for the first time, evolutionary relation between previously overlooked nitroreductases from Viridiplantae including Chlorophyta, Bryophyta, Marchantiophyta, Lycopodiopsida, and Spermatophyta. Results of the phylogenetic tree reconstruction and structural models’ comparison suggest that green algae and cyanobacteria are the closest relatives for the modern plant nitroreductases. Conserved active sites, required for the co-factor binding, and chloroplastic/mitochondrial localizations imply primary physiological function in the oxidative stress response. In total, results of this study provide the first theoretical background for the future research of the Viridiplantae-delivered nitroreductases and discuss prospective areas for their practical application.

## Supplementary information

**Additional file 1: Supplementary Table 1.** Accessions (UniProt) list of bacterial, algal, and plant proteins, identified in this study. Sequences were used for phylogenetic tree reconstruction (Supplementary Figure 1 and 2). Taxonomy was simplified. Domains are named according to the Pfam database [21]. Presence of both N- and C-terminal Nitroreductase domains are depicted as “x2 domain”. Predicted subcellular localization (TargetP): C – chloroplast, M – mitochondria, S – secreted, “-” – not defined.

**Additional file 2: Supplementary Figure 1.** Phylogeny estimation of identified nitroreductase domain-containing proteins. The Neighbor-Joining method and JTT model were used; 1000 bootstrap replicates. Only branches with bootstrap value >50 are shown.

**Additional file 3: Supplementary Figure 2.** Phylogeny estimation of identified nitroreductase domain-containing proteins. The Maximum Likelihood method and LG model were used; 1000 bootstrap replicates. Only branches with bootstrap value >50 are shown.

## Data Availability

Available upon request

## Abbreviations

FMN: Flavin mononucleotide
FAD: Flavin adenine dinucleotide
NADH: Nicotinamide adenine dinucleotide
NADPH: Nicotinamide adenine dinucleotide phosphate
BLAST: Basic local alignment search tool
NCBI: National Center for Biotechnology Information
JTT: Jones-Taylor-Thornton
