# Supplementary figures and images for: Evolutionary aspects of the Viridiplantae nitroreductases
Source: J Genet Eng Biotechnol. 2020 Oct 6;18:60. doi: 10.1186/s43141-020-00073-3 (PMC7538488; doi:10.1186/s43141-020-00073-3)

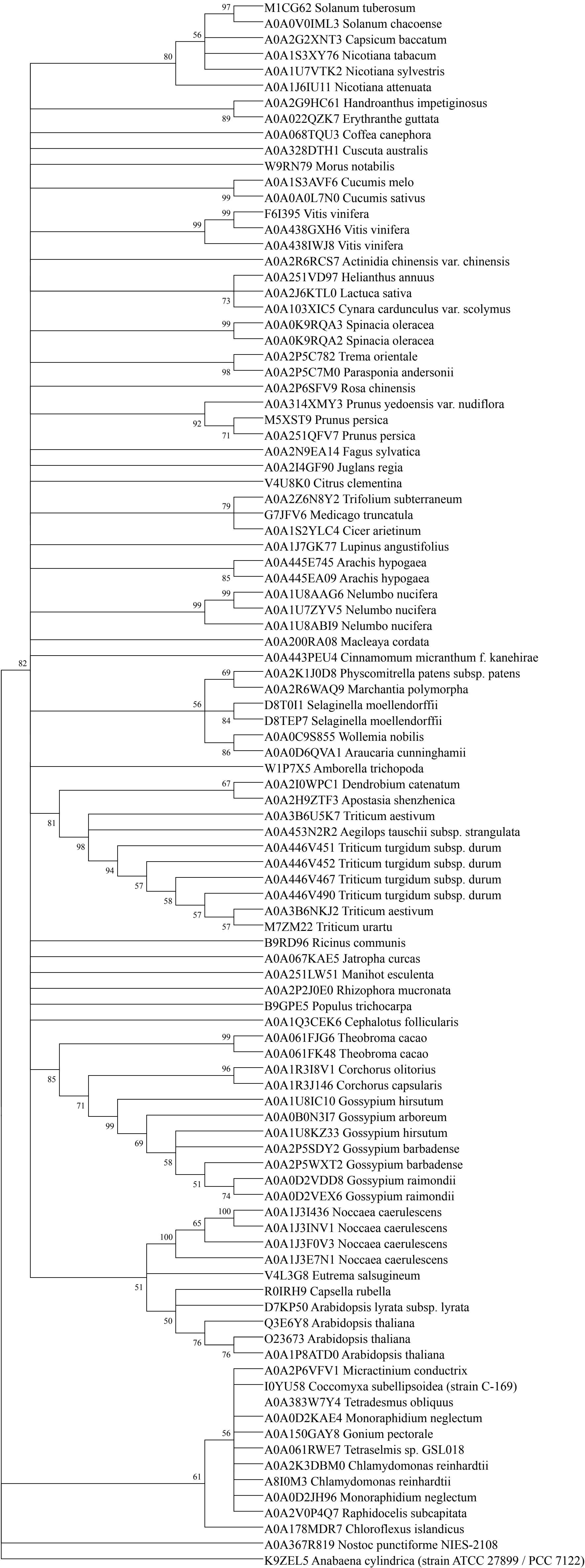

Green algae

Chloroflexi

Cyanobacteria

Supplement: Supplementary file 2 — Additional file 2: Supplementary Figure 1. Phylogeny estimation of identified nitroreductase domain-containing proteins. The Neighbor-Joining method and JTT model were used; 1000 bootstrap replicates. Only branches with bootstrap value >50 are shown. [file 43141_2020_73_MOESM2_ESM.pdf]

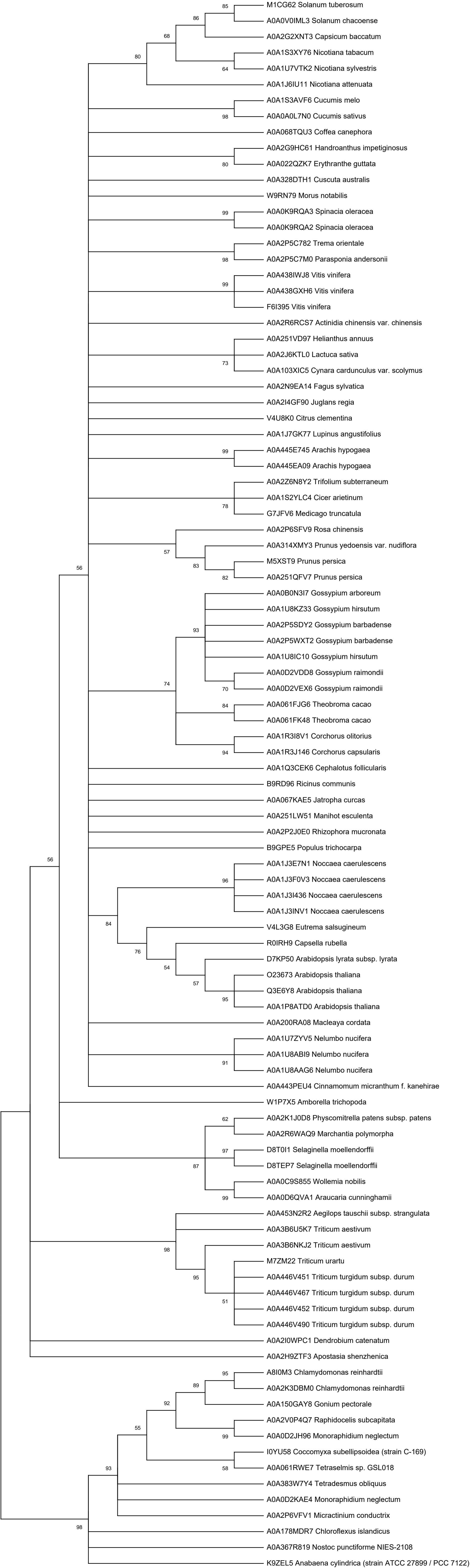

Supplement: Supplementary file 3 — Additional file 3: Supplementary Figure 2. Phylogeny estimation of identified nitroreductase domain-containing proteins. The Maximum Likelihood method and LG model were used; 1000 bootstrap replicates. Only branches with bootstrap value >50 are shown. [file 43141_2020_73_MOESM3_ESM.pdf]
